# One Beam, Dual Insights: Simultaneous Chemical and Structural Changes in Nanopatterned Ceria under Reaction Conditions

**DOI:** 10.1021/acs.jpclett.6c00039

**Published:** 2026-02-19

**Authors:** Adva Ben Yaacov, Maximilian Jaugstetter, Heath Kersell, Ora Simcha Bitton, Miquel B. Salmeron, Slavomír Nemšák, Baran Eren

**Affiliations:** † Department of Chemical and Biological Physics, 34976 Weizmann Institute of Science, 234 Herzl Street, 76100 Rehovot, Israel; ‡ Chemical Sciences Division, 1666 Lawrence Berkeley National Laboratory, Berkeley, California 94720, United States; § Advanced Light Source, Lawrence Berkeley National Laboratory, Berkeley, California 94720, United States; ∥ Chemical Research Support, Weizmann Institute of Science, 234 Herzl Street, 76100 Rehovot, Israel

## Abstract

Ceria’s interaction with hydrogen can proceed through multiple chemical forms (hydride, hydroxyl, and oxyhydroxide-like), with consequences for the oxidation state, density, and morphology that are rarely tracked in the same evolving state. Here we show that under mild H_2_ (and H_2_ and CO_2_) environments nanopatterned ceria undergoes oxidation-state changes accompanied by hydrogen incorporation that increases the effective electron density, establishing the following order: CeO_2_H_*y*_ > CeO_2_ > CeO_2–*x*_H_*y*_ > CeO_2–*x*_. In parallel, the surface roughens in a chemically specific manner, with the largest changes coinciding with conditions where incorporated hydrogen is driven to react with oxygen supplied either by air exposure between experiments or by added CO_2_. We obtained these insights by using a single X-ray beam to simultaneously perform ambient-pressure X-ray photoelectron spectroscopy and grazing-incidence X-ray scattering on the same sample spot. Single-mode measurements can miss key ceria–H_2_ transformations relevant to optimizing ceria-based hydrogenation catalysts and supports.

## Graphical abstract

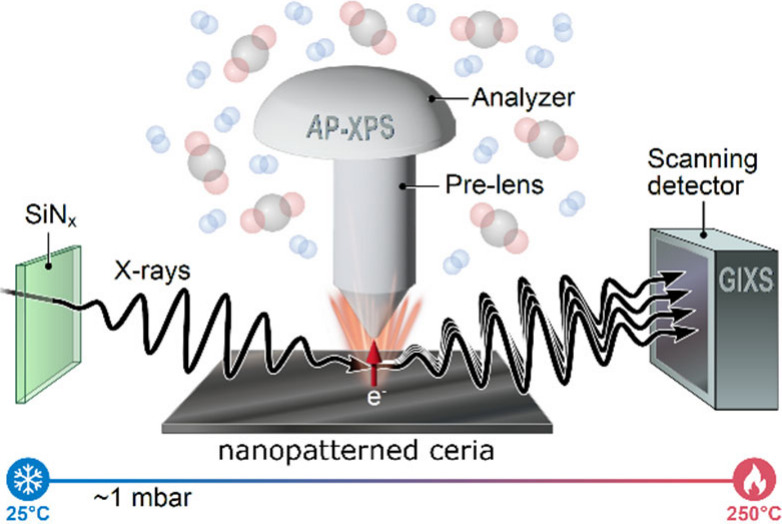

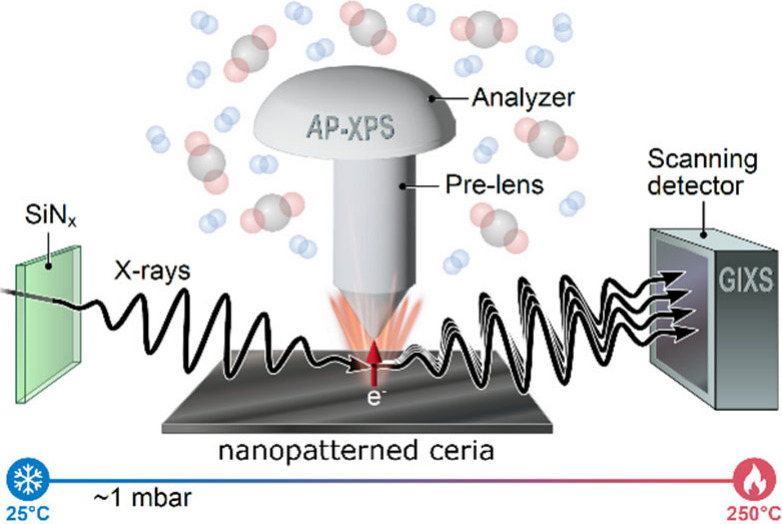

With its labile oxidation state, abundant oxygen vacancies, and strong capacity to activate, shuttle, and store hydrogen, ceria offers a dynamic, defect-rich surface that enables efficient and selective hydrogenation pathways.^1,2^ Understanding catalytic hydrogenation on reducible oxides such as ceria requires linking surface chemistry to the structural response in the same state. Here, a single X-ray beam provides simultaneous, coregistered chemical and structural information,^3^ eliminating run-to-run variability and enabling direct chemistry–structure correlations. The combined measurements reveal changes in the oxidation state, hydrogen incorporation that increases the electron density (i.e., hydride/oxyhydroxide), and surface roughening under H_2_ and under H_2_ and CO_2_. Spectroscopy alone would miss these closely connected changes.^4^

Beyond demonstrating a coregistered spectroscopy and scattering workflow (tailored sample preparation, measurements, and data fitting), our work provides three materials-related outcomes on ceria–H_2_ interaction under reaction conditions. First, hydrogen incorporation can increase ceria’s electron density, yielding a reproducible hierarchy (CeO_2_H_*y*_ > CeO_2_ > CeO_2–*x*_H_*y*_ > CeO_2–*x*_) that separates vacancy-associated and interstitial/subsurface hydrogen uptake. Second, for defect-rich polycrystalline ceria, thin films resemble subsurface hydrogen-containing ceria (oxyhydroxide-like) already at 200–300 °C, indicating that microstructure and defect density tune the onset of hydrogen incorporation. Third, the roughening is chemically specific. It is largest when incorporated hydrogen is subsequently driven to react with oxygen supplied by air or CO_2_, and its reversibility differs between conditions with only H_2_ and those with H_2_ and CO_2_.

We prepared nanopatterned ceria using electron-beam (e-beam) lithography and e-beam evaporation (Methods). The shape of the model material was chosen as a collection of randomly positioned ceria cylinders (average height of 24 nm and average diameter of 73 nm), verified by atomic force microscopy (AFM) images (Section S1 of the Supporting Information). The random positioning of the cylinders is essential, since only the shape of the cylinders via their form factor contributes to the scattering signal, as opposed to the structure factor in the form of high-intensity Bragg maxima that otherwise dominate the signal in periodically ordered systems.^5^ Hence, any changes in the scattering data can be attributed to the collective change in the shape, roughness, or density of the ceria cylinders.

Two sets of experiments were performed, the first one to understand H_2_–ceria interaction and the second one with CO_2_ added to the gas mixture, resembling the CO_2_ hydrogenation reaction (Methods).^6,7^ The same sample was used for both sets of experiments, starting with H_2_ exposure experiments and finishing with experiments with CO_2_ and H_2_. Since the sample was carried through air before and between experiments, a cleaning protocol was employed (Methods). We performed simultaneous chemically sensitive ambient-pressure X-ray photoelectron spectroscopy (AP-XPS) and structure-sensitive grazing-incidence X-ray scattering (GIXS) measurements to understand the chemical state and structure in each experiment.^8^

Panels A–C of Figure 1 show examples of Ce 4d and O 1s regions of the AP-XPS spectra and GIXS images. AP-XPS provides detailed information on surface chemistry, including the percentage of different Ce cations (via Ce 4d spectra) and the OH coverage (via O 1s spectra) on the surface (Section S2). Changing trends in surface chemistry with changing conditions are presented in panels i and ii of Figure 2. In order to interpret the GIXS data, we took line profiles at **q**_r_ = 0 along the scattering plane (Figure 1D), which contains out-of-plane information in real space (Section S3). The information in the line profiles can be divided into three regions: the low-**q**_*z*_ region (not used in our analysis), the central region, and the high-**q**_*z*_ region. The central region governs the changes in height of the ceria cylinders,^9^ whereas the decay of the signal with an increase in **q**_*z*_ is related to the roughness of the surface.^10^ By using models whose form factors along the **q**_r_ = 0 line profile match those of the acquired data (Sections S3 and S5), we extracted four parameters: cylinder height (Section S6), scattering length density (SLD), which increases with the electron density of the scattering material (Section S6), and roughness (Figure 2iv). The roughness corresponds to the effective surface-fractal dimension (Section S5) and, hence, is unitless in our analysis. The scattering images are also used to extract the X-ray reflection critical angle (Section S4), which depends on the density of the material. We present this as a relative critical angle, which is the difference between the critical angles for ceria and the silicon substrate (Figure 2iii), since the absolute angles are influenced by the sample (mis)­alignment. We assume that the amount and effect of contaminants on ceria and silicon are the same and, hence, cancel when the critical angle is presented as a relative quantity.

**Figure 1:**
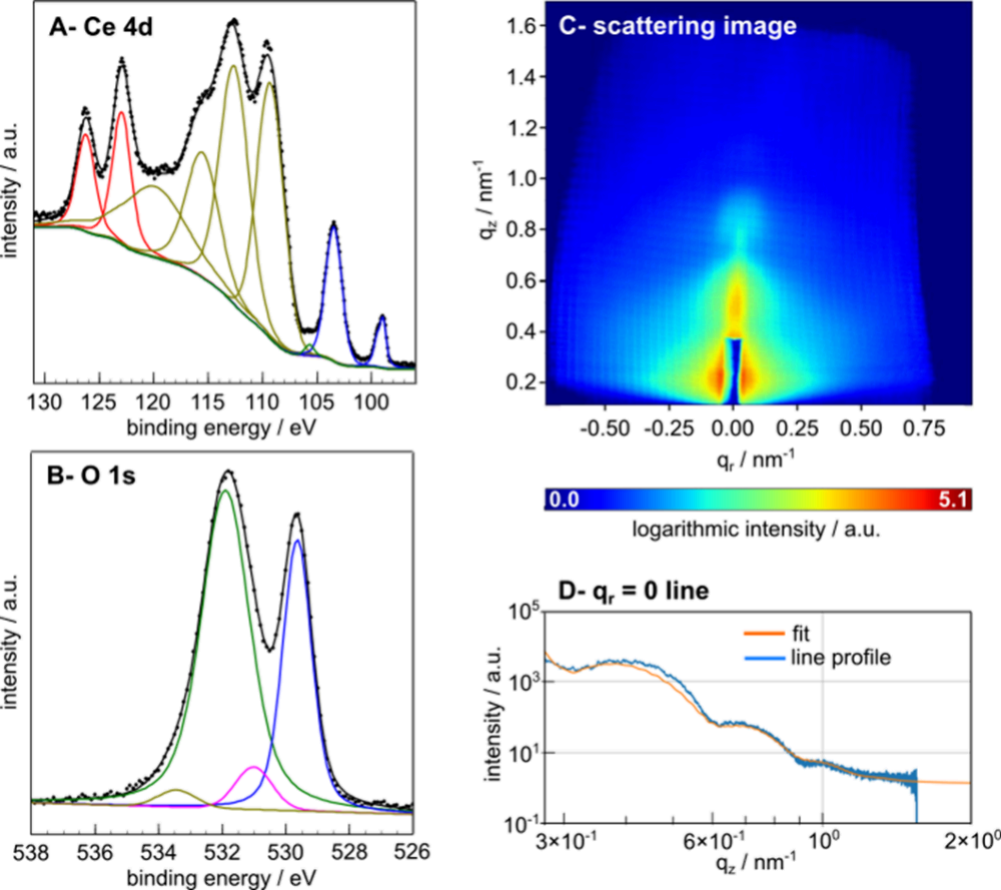
(A) Ce 4d spectrum. Red peaks are due to Ce^4+^ cations. The weak green peak is due to Ce^3+^ cations. Blue peaks are due to the Si substrate with the native oxide. Yellow features are due to both Ce^4+^ and Ce^3+^ cations. (B) O 1s spectrum. The blue peak is due to ceria. The magenta peak is due to hydroxyls. The green peak is due to the native oxide of the Si substrate. The yellow peak is due to water. Further explanations of the peak positions can be found in Section S2. (C) GIXS image. (D) Line profile along **q**_r_ = 0 extracted from the GIXS image in panel C, along with a fit. Both examples are from the second set of measurements prior to any treatment or gas dosing.

**Figure 2:**
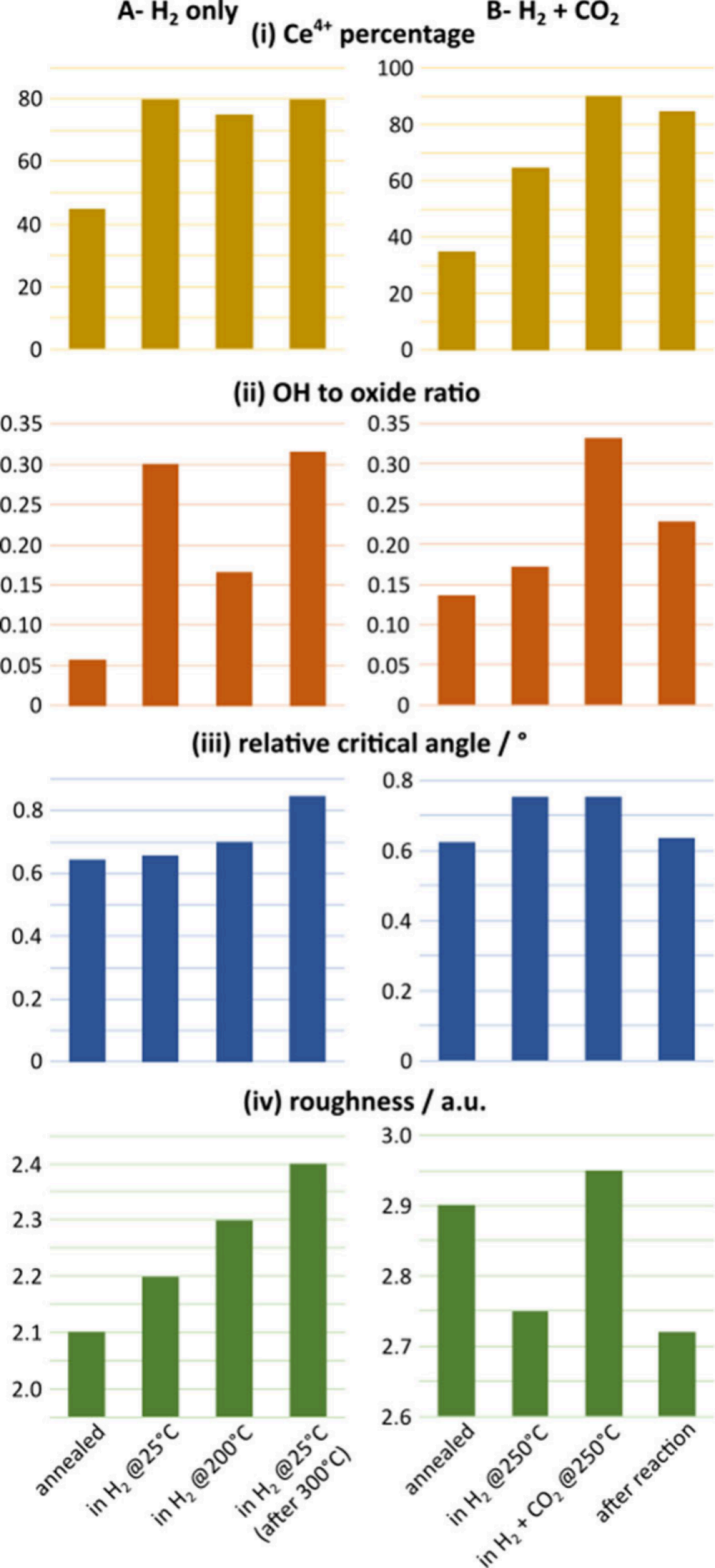
(A) H_2_ experiment and (B) H_2_ and CO_2_ experiment: (i) chemical state of ceria, obtained from the Ce 4d spectra; (ii) OH:ceria ratio obtained from the O 1s spectra, (iii) relative critical angle obtained from GIXS images, and (iv) roughness of ceria cylinders, obtained from the **q**_r_ = 0 line profile.

Reduced ceria is a material that is rich in oxygen vacancies, which is believed to be active in dissociating H_2_ already at room temperature.^4,11–15^ H_2_ activation on ceria can proceed via homolytic hydride formation (oxidizes reduced ceria), homolytic hydroxyl formation (reduces stoichiometric ceria), or heterolytic pathways (no change in oxidation state), depending on the vacancy density and conditions.^4,11–15^ In practice, all of these processes take place at different rates. Figure 2i shows the changes in the Ce^4+^ percentage in the surface layers (the rest of it being Ce^3+^) under different experimental conditions. These trends were obtained using a linear combination of reference Ce 4d XPS spectra. Initially, the ceria surface appears to be quite reduced due to the thermal treatment in vacuum at 450 °C. The oxidation of ceria in H_2_ at 25 °C due to hydride formation (CeO_2–*x*_H_*y*_, where *x* > *y*) and slight reduction thereafter at 200 °C due to hydroxyl formation is visible in Figure 2A-i, as also discussed in detail in our previous AP-XPS study.^4^ The ceria surface is oxidized further once CO_2_ is added to the gas mixture (Figure 2A-i). This is due to the dissociative adsorption of CO_2_ at oxygen vacancies and other undercoordinated defect sites of the surface, which is feasible even without thermal activation at such CO_2_ partial pressures.^16,17^

The OH:oxide intensity ratio obtained from the O 1s spectral fitting (Figure 2A-ii) suggests that the reduced ceria surface is covered with hydroxyls in H_2_ at 25 °C, which can be attributed to adsorption of H_2_O impurities in H_2_ onto the surface.^4^ While hydroxyls are homolytically and heterolytically generated at 200–250 °C, they also start desorbing; hence, their coverage appears to be lower than at 25 °C. At 300 °C, the surface hydroxyl coverage is expected to be very low due to desorption,^11^ but the detected amount of OH species under such conditions is nevertheless slightly higher compared to vacuum conditions (Figure S2b), which could be related to the subsurface of ceria in the form of oxyhydroxides. Consistent with this interpretation, the fitted oxide component shows a distinct broadening relative to the vacuum-annealed reference, supporting the presence of multiple oxygen environments beyond simple surface OH adsorption (Section S2). Oxyhydroxide (CeO_2_H_*y*_, where *y* < 1) formation in the subsurface/bulk was reported to take place at ≥327 °C.^18–21^ In the present case, the defective nature of the thin films allowing bulk diffusion could facilitate their formation even at 200–300 °C. The addition of CO_2_ to the gas mixture increases the relative intensity of the peak assigned to OH, but this increase is caused by the formation of oxygen-containing CO_2_ and H_2_ reaction intermediates/products (e.g., formate) on the surface (Figure S2e).^6,22^ Such oxygenated species and OH have overlapping peaks in the O 1s spectra, but there is also evidence of them in the C 1s spectra (Figure S2f).

The formation of hydrides and oxyhydroxides, as well as oxidation back into CeO_2_ through dissociation of CO_2_, should cause lattice expansions and contractions.^19–21^ These are not reflected in the form of changes in the height of the ceria cylinders, which changes by only ±0.1 nm between measurements (Section S6) and falls below our measurement accuracy. We should also underline that the GIXS method can detect significant changes in height when they are present; for example, the thermal treatment of the pristine samples in high vacuum decreases the height of the cylinders by ∼1 nm (Section S7). We believe that the lack of external volumetric changes is due to the defective nature of the polycrystalline thin films, as they are typically porous, have packing densities of less than 70%, and are rich in structural defects.^23^ Therefore, the lattice expansions cannot be measured externally; rather, they exhibit expansion by increasing the overall density and filling the internal pores and voids. In other words, for defect-rich nanostructured ceria, the most sensitive structural signature of H incorporation in our experiments is densification (e.g., subsurface uptake) rather than a macroscopic height change; i.e., the evidence for hydride and oxyhydroxide formation should come from density variations. In the first set (Figure 2A), there is a slight increase in the relative critical angle in H_2_ at 25 °C, due to hydride formation in the oxygen vacancies (higher electron density). We observe a further increase at 200 °C, which is a consequence of oxyhydroxide formation (hydrogen starting to occupy interstitial sites in addition to vacancies). Prior to cooling back to 25 °C, the sample was first heated to 300 °C, cooled to 200 °C, and then further cooled to 25 °C, each step taking around 75 min. This means that more of these oxyhydroxides formed in the bulk, increasing the overall electron density. In the second set (Figure 2B), a similar phenomenon takes place in H_2_ at 250 °C. It is reversed slightly as the oxyhydroxide starts converting to CeO_2_ with CO_2_ producing atomic oxygen that replaces OH^–^ in the bulk. As the sample is cooled, this effect continues to take place. Accordingly, the electron density relationship of different ceria species formed during the process is as follows: CeO_2_H_*y*_ > CeO_2_ > CeO_2–*x*_H_*y*_ > CeO_2–*x*_. Reduction of CeO_2_ to CeO_2–*x*_ removes oxygen (reducing the electron count), but it also typically expands the lattice,^24^ increasing the unit cell volume; thereby, the electron density decreases even further. Our results further show that hydrogen, at either the vacancy sites or the interstitial sites, increases the electron density. Such useful information eludes pure spectroscopy studies.

As a technical note, a mixture of stoichiometric oxide and hydroxide would yield an effective electron density comparable to that of an oxyhydroxide and would therefore appear similarly in relative critical angle and SLD metrics. Because in the literature bulk hydroxide formation under H_2_ has not been reported for ceria, we excluded this scenario from consideration. This ambiguity does not change the central conclusion that hydrogen incorporation increases the effective electron density under these conditions.

Adsorbate-driven reorganization of the surface atoms due to the changing environment is often associated with metals due to their low surface diffusion barriers.^25,26^ Although surface atoms can reorganize to maximize bonding with adsorbates when the energy gained from stronger adsorbate–surface interactions exceeds the cost of breaking surface–surface bonds, such reorganization may be kinetically hindered.^27,28^ In particular for strongly ionically and covalently bonded materials, such as oxides, higher temperatures might be needed to observe such surface restructuring. Weak coordination of the outermost atoms in an evaporated thin film is expected to facilitate such surface modification. Figure 2A shows that even at 25 °C, reaction with H_2_ roughens the ceria surface. As the temperature is increased first to 200 °C and then to 300 °C, further roughening takes place even after the temperature is reduced back to 25 °C. Both surface hydroxyls and surface hydrogen produced by the homolytic and heterolytic reaction pathways could be triggering the adsorbate-driven roughening even at low equilibrium coverages. At higher temperatures, removal of oxygen from the surface either thermally or via water vapor formation also contributes to roughening of the surface.^29,30^

Even though the estimated roughness has arbitrary units and is not directly comparable between the two sets of experiments, the following conclusions can be drawn by using the observed trends. Unlike in the first set, we do not observe a roughening effect of H_2_ treatment in the second set, but rather slight smoothening (Figure 2B). This implies that the initial surface is rougher in the second set than in the previous case; it is also more hydroxylated. This could be because of the incorporated hydrogen reacting with oxygen in air between the two measurement sets, leaving an even more porous structure. Once CO_2_ is included in the gas mixture, the roughness increases. In this case, either CO, atomic oxygen, or the oxygenated hydrocarbons that form upon CO_2_ dissociation adsorb strongly on the weakly coordinated sites; therefore, surface roughening becomes energetically favored. Evacuating the gases reverses the roughening taking place under reaction conditions (set 2), but it does not revert roughening taking place due to hydrogen incorporation (set 1).

Both hydrides and oxyhydroxides form under the mild pressure and temperature conditions chosen in this study, which both increase the electron density. We thereby establish the electron density (and, in fact, atomic density since volumetric changes are insignificant) relationship of various ceria species formed under each condition as CeO_2_H_*y*_ > CeO_2_ > CeO_2–*x*_H_*y*_ > CeO_2–*x*_. Another important outcome is that an increase in roughness always occurs when the incorporated hydrogen reacts with oxygen atoms, supplied by either O_2_ in air (between two sets) or CO_2_ gas (in set 2). Interaction with hydrogen, without any other gas, also causes adsorbate-driven surface roughening but to a lesser extent. The evolving state of ceria observed in this work is summarized in Figure 3. Ceria can play a critical role in the CO_2_ hydrogenation reaction.^31,32^ Access to such a variety of data from “materials in action” in a single experiment can have far-reaching implications, since the hydrogen type,^33^ the roughness, and the chemical state of the surface, which all depend on the reactive environment, directly affect the material performance. We expect our method to be adapted to applied catalysis research in the near future, using shape-controlled nanoparticles in place of e-beam-grown model materials.

**Figure 3:**
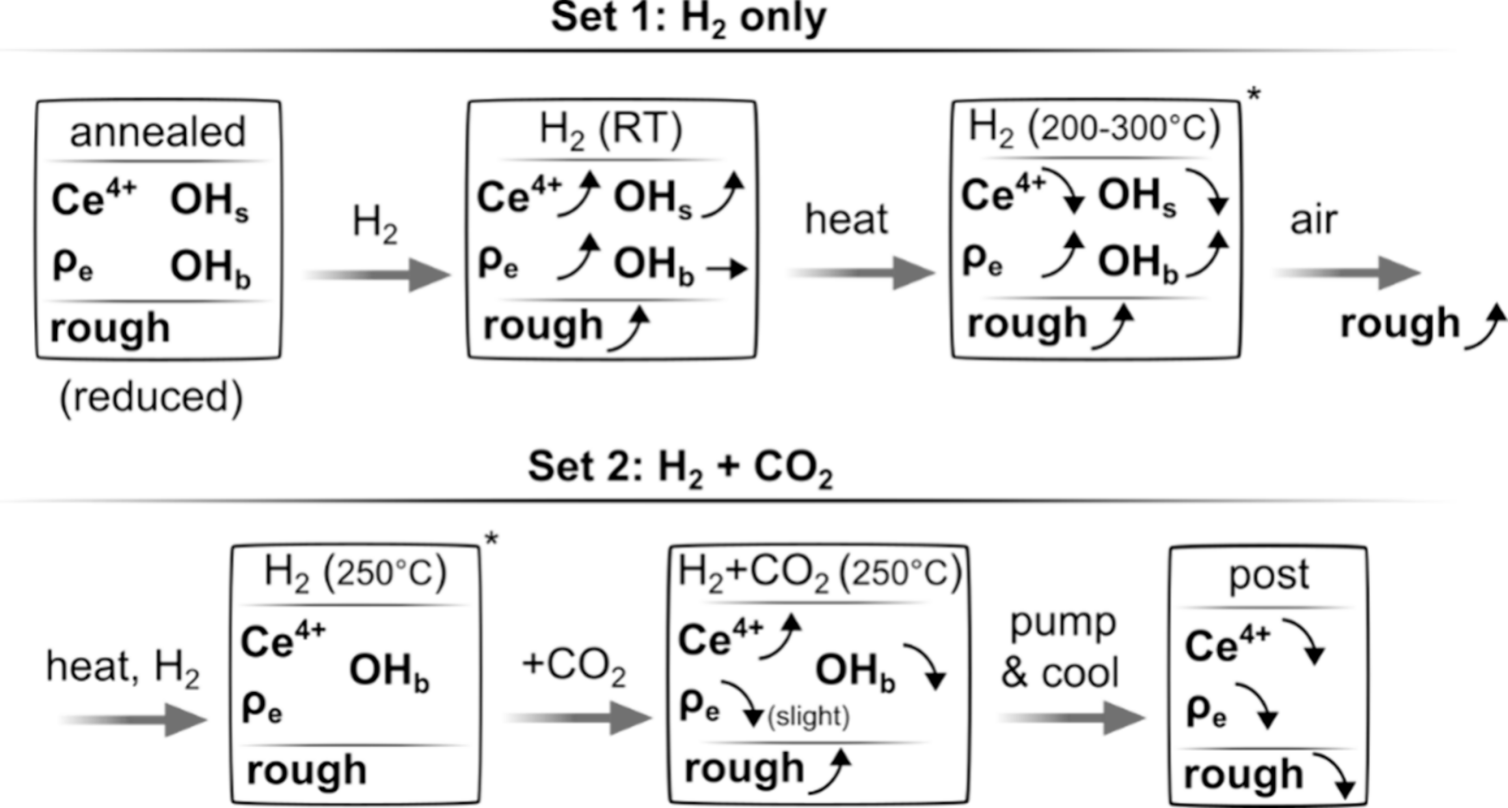
Schematic summary of the changes in ceria based on interpretation of our observations in two experimental sets. “rough” stands for roughness, OH_s_ surface hydroxyls, and OH_b_ bulk species associated with oxyhydroxide. Within each set, arrows denote the direction of change relative to the immediately preceding condition and the first box serves as the reference state for that set. The states marked with an asterisk in the upper and lower rows correspond to closely comparable conditions and are included to provide a convenient reference for linking the two sets.

## Methods

*Sample Preparation*. Samples were prepared by e-beam lithography and e-beam evaporation as follows. PMMA 950K A2 was spin coated on heavily doped Si substrates (necessary for electrical conductance during experiments with X-rays to minimize charging) at a speed of 5000 rpm for 45 s to achieve a thickness of 60 nm, followed by baking at 180 °C for 90 s. The PMMA-coated substrates were then loaded into a Raith E-line Plus e-beam lithography system, and the PMMA was exposed at an accelerating voltage of 30 kV and an aperture size of 20 μm, yielding a beam current of 120 pA. The design was exposed in “single dots” mode, in which every cylinder is formed by exposing a pixel. The disc diameter is determined by the dwell time that the e-beam is maintained at a certain pixel. We used a dose of 1 pC per pixel (dwell time adjusted accordingly), resulting in cylinders with a diameter of ∼70 nm. To remove the exposed PMMA, substrates were developed in a 1:3 methyl isobutyl ketone/isopropyl alcohol mixture for 30 s, followed by immersion in a stopper (isopropyl alcohol) for 30 s and drying with a nitrogen flow. An e-beam evaporator (Odem Scientific Applications Ltd.) was used for depositing ∼25 nm thick CeO_2_. The substrates were kept at 25 °C during the deposition. Following deposition, the substrates were immersed in a PG remover heated to 85 °C for a few minutes to lift off the PMMA. The substrates were then sonicated for an additional few minutes to ensure full removal of the PMMA.

The design of randomly dispersed pixels was prepared in MATLAB software and then imported into the Raith E-line Plus system. We kept the distance between pixels to >40 nm to minimize the overlap between the cylinders.

X-ray diffraction patterns of the prepared samples predominantly exhibit the (220) peak; i.e., the sample is textured along the (110) direction. No prominent differences in the diffraction patterns can be observed before and after the experiments with H_2_ or experiments with H_2_ and CO_2_, as in both cases the bulk chemistry and structure are likely to be governed by the oxidation taking place during storage in air.

*AP-XPS and GIXS Measurements*. Combined AP-XPS and GIXS measurements were performed at beamline 11.0.2 of the Advanced Light Source (ALS) in Berkeley.^8^ The photon energy (*E_hυ_*) was fixed at 1000 eV for both core-level XPS and X-ray scattering measurements. The attenuation length/information depth of the XPS measurements is approximated by photoelectron inelastic mean free path, which is around 2 nm for Ce 4d.

The end station is equipped with a SPECS hemispherical electron analyzer capable of operating at gas pressures of ∼10 Torr. All XPS spectra were acquired with a pass energy of 20 eV, resulting in a total energy resolution (analyzer and beamline) of ∼0.5 eV. The electron detection angle was 15° off normal.

The X-ray scattering signal was collected using an Andor Ikon-L CCD detector. The X-ray camera is mounted on a biaxial rotating manipulator covering ±12° in-plane and 24° out-of-plane scattering angles, corresponding to a scattering vector **q** range of ±1.2 and ±2.4 nm^–1^, respectively. The detector is separated from the reaction chamber by a large-area Si_3_N_4_ X-ray window matching the size of the camera chip (27 mm × 27 mm).

The incidence angle of the X-ray beam with respect to the sample surface was close to the critical angle of the Si substrate (1.7° at 1000 eV).^34^

The base pressure of the measurement chamber was below 1 × 10^–8^ Torr. The gas handling system comprised of vacuum manifold and reaction gases (H_2_, CO_2_, and O_2_) were introduced through individual lines connected to the chamber by manual leak valves. Pressures of less than 1 × 10^–4^ Torr were measured using an ion gauge, while higher (<0.1 Torr) pressures were determined by a Pirani gauge.

Two sets of experiments were performed, the first one to understand H_2_–ceria interaction and the second with CO_2_ included in the gas mixture in order to understand its additional effects. The same sample was used for both sets of experiments, one after another, after storage in the air for a couple of days. In each case, the sample was first annealed to 450 °C in high vacuum and cooled afterward to 25 °C in order to remove contaminants from storage in air. Subsequent experimental conditions are as follows: (I) as annealed; (II) in 100 mTorr of H_2_, sample at 25 °C; (III) in 100 mTorr of H_2_, sample heated to 200 °C; (IV) in 100 mTorr of H_2_, sample cooled to 25 °C after heating to 300 °C in H_2_ for the first set; (I) as annealed; (II) in 100 mTorr H_2_, sample at 250 °C; (III) in the presence of 66 mTorr of H_2_ and 45 mTorr, sample of 250 °C; (IV) sample cooled back to 25 °C after both gases are pumped out of the chamber in the second set.

## Supplementary Material



## Notes

AFM images of the sample, all of the XPS spectra and the details of XPS fitting, all of the scattering images and the details of GIXS fitting, details of X-ray reflection critical angle analysis, explanation of the scattering models and used formulas and sanity checks, further analysis of the observed trends, and discussion of the effects of initial annealing (PDF)
