# Supplementary material for: One Beam, Dual Insights: Simultaneous Chemical and Structural Changes in Nanopatterned Ceria under Reaction Conditions
Source: J Phys Chem Lett. 2026 Feb 19;17(9):2729–34. doi: 10.1021/acs.jpclett.6c00039 (PMC12969358; doi:10.1021/acs.jpclett.6c00039)
Supplement: Supplementary file 1 [file jz6c00039_si_001.pdf]

Supplementary Information for

'One Beam, Dual Insights: Simultaneous Chemical and Structural Changes in Nanopatterned Ceria Under Reaction Conditions'

Adva Ben Yaacov[1], Maximilian Jaugstetter[2], Heath Kersell[3], Ora Simcha Bitton[4], Miquel B. Salmeron[2], Slavomír Nemšák[3], Baran Eren[1]

[1]Department of Chemical and Biological Physics, Weizmann Institute of Science, 234 Herzl Street, 76100 Rehovot, Israel

[2]Materials Sciences Division, Lawrence Berkeley National Laboratory, Berkeley, California 94720, United States

[3]Advanced Light Source, Lawrence Berkeley National Laboratory, Berkeley, California 94720, United States

[4]Chemical Research Support, Weizmann Institute of Science, 234 Herzl Street, 76100 Rehovot, Israel

Section S1- AFM images of the sample

Atomic force microscopy (AFM) images were obtained after preparation of the sample. An example is shown in Fig. S1. We used amplitude-modulated intermittent mode AFM and recorded both topography and phase contrast channels. The AFM image represents a small portion of the sample area, as the total sample area is larger than the size of the X-ray beam.

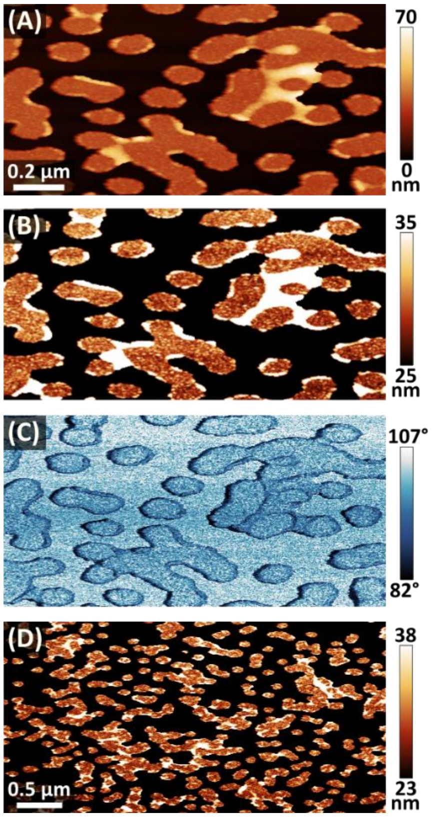

Fig. S1. AFM images of the sample. **(a)** Full z-scale topography image. **(b)** Reduced z-scale topography image to show the roughness of the ceria cylinders. **(c)** Phase contrast image, showing different phase shifts (with respect to the reference phase of the controller) between the substrate (brightest features), polymer residues (darkest features), and the ceria regions (in-between brightness). **(d)** Larger xy-scale topography image with reduced z-scale.

Section S2 - Details of AP-XPS fitting

Fig. S2 shows all the collected XPS spectra and fits. The full width at half maximum (FWHM) and relative positions of all the peaks were constrained to be consistent between the spectra acquired at different conditions.

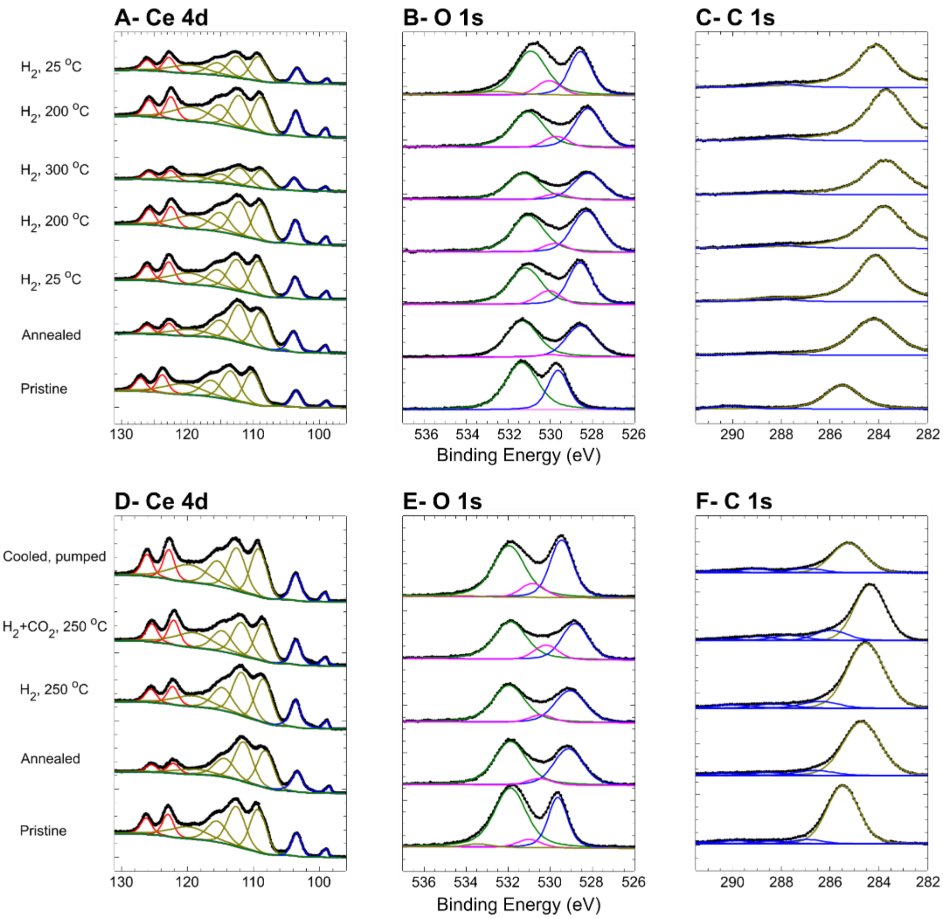

Fig. S2. AP-XPS spectra collected at different conditions with fits. **(a, d)** Ce 4d – Si 2p region. Red peaks are due to the Ce^{4+} cations, the low-intensity green peak is due to the Ce^{3+} cations, yellow peaks are due to both cations, and the blue peaks are due to the silicon substrate and its native oxide. **(b, e)** O 1s region. Blue peak is due to ceria, magenta peak is due to hydroxyls, green peak is due to silica, and yellow peak is due to molecular water. **(c, f)** C 1s region, mainly consisting of the hydrocarbon peak. Some features appear at higher binding energies for the H_2+CO_2 case due to the production of various oxygenated hydrocarbons.

Ce 4d

We measured the 95-140 eV spectral region which includes the Ce 4d and Si 2p core-levels (Fig. S2a). The fitting of this region was done in a similar way to our previous paper (S1): A combination of nine Gaussian-Lorentzian lineshapes were employed after Shirley background subtraction to fit the spectral region. Seven of them are related to the Ce 4d core-level, and two are related to the Si 2p core-level (in blue). The first Si-related Gaussian-Lorentzian peak is positioned at the second lowest binding energy, which is due to the native oxide of the Si substrate. The other Si-related peak is due to unoxidized Si that is underneath the native oxide, which appears at the lowest binding energy.

Features related to the Ce 4d are the following: two Gaussian-Lorentzian peaks that are highest binding energies which are due to the Ce^{4+} ions (in red). The spin-orbit splitting between these two peaks is fixed to 3.2-3.3 eV during fitting (S2-S3). A low-intensity Gaussian-Lorentzian peak at the lowest binding energy, excluding silica-related peaks, which is due to the Ce^{3+} ions (in green) (S4). All the other features in between the red and green peaks are due to both Ce^{3+} and Ce^{4+} ions (in yellow). The relative intensities of the yellow features clearly depend on the oxidation state of the material, but their origin is disputed in the literature (S2,S5).

While the positions of the Si peaks are rather constant at different reaction conditions, positions of the features related to ceria show dependence on the measurement conditions. We also observe a similar trend in the position of some of the peaks in O 1s and C 1s regions, except for those related to SiO_2. This is due to the semiconducting nature of ceria and therefore band bending. The relative positions of these features in the Ce 4d – Si 2p region were constraint during fitting to account for this effect.

Because of the overlapping Ce^{3+} and Ce^{4+} features (i.e., the yellow features), it is not possible to quantify the concentration of cations from fitting. However, we can estimate it by using the following approach: We take the difference between S_actual and b*S_oxidized, where S_oxidized is a reference spectrum taken after full oxidation in O_2, and b is chosen such that the red peaks are in the noise level in the difference spectra. This way, b gives us the percentage of Ce^{4+} in ceria. The outcomes are presented in Fig. 2-i.

O 1s

In pristine conditions, the O 1s region consists of four Gaussian-Lorentzian peaks (Fig. S2b). The most prominent peaks are due to oxides: The lower binding energy peak due to ceria and the higher binding energy peak due to silica, which are shown with blue and green colors, respectively. The magenta peak which appears in between them is due to OH that are both on the surface and in the bulk. In some conditions, at 25 °C, there is a very small peak at the highest binding energy which is due to molecular water (in yellow).

In the literature, the positions of the peaks are assigned as follows: In (S2), Ce^{4+} cations produced a peak at 530.4 eV while the Ce^{3+} cations produced a peak that is at 0.3 eV higher binding energy and the hydroxyls on reduced ceria produced a peak at around 2.1 eV higher binding energy. In (S6), the peak due to the Ce^{3+} cations was reported at 529.8 eV, and the peak due to hydroxyls on reduced ceria was reported at approximately 2 eV higher in energy. In (S3), Ce^{4+} and Ce^{3+} cations were found to have peaks at 529.4 eV and 530.2 eV, respectively. The hydroxyl peak was observed at 531.7 eV. According to (S7), Ce^{4+} cations produce a peak at 529.4 eV, reduced ceria produces a peak in the range of 529.8-530.0 eV, and hydroxyl groups on reduced ceria produce peaks between 532.0-532.5 eV. According to (S5), Ce^{4+} cations are expected to exhibit a peak at 530.4 eV. Reduced ceria should have a peak approximately 0.3 eV higher than this peak, while the hydroxyls on reduced ceria should produce a peak around 2.1 eV above the first peak. (S8) suggests that oxygen anions adjacent to Ce^{4+} cations have a peak with a binding energy of 529.3 eV for CeO_2 and 529.7 eV for CeO_{2-x}. It is claimed that oxygen anions next to Ce^{3+} cations in CeO_{2-x} exhibit a peak with a binding energy of 532.0 eV. The OH/H_2O peak was reported to appear at 530.9 eV for CeO_2 and 531.7-532.0 eV for CeO_{2-x}.

As shown in the above discussion, the literature values related to the oxygen that is neighboring different Ce cations are highly disputed. An important factor here is that as in the case of the Ce 4d spectra, their positions change with the experimental conditions (which could explain the discrepancy in the literature) due to semiconducting nature of ceria and band bending.

We used one umbrella peak to for oxygen anions adjacent to both Ce^{4+} and Ce^{3+} cations. We do think that these two peaks are too close to each other to deconvolute them, especially with unreferenced binding energies and when band bending is present.

The magenta peaks that are assigned to OH groups also have some contribution from the oxygenated hydrocarbons that are either some form of contaminants or form during the CO_2 hydrogenation reaction. In fact, this peak clearly increases in intensity when CO_2 is added into the gas mixture. In some conditions, the decrease in normalized hydroxyl intensity (Fig. S3b) might be related to the oxidation of the surface, i.e., reduced ceria surfaces are covered more with hydroxyls than oxidized ceria surfaces. We discussed this in more detail in (S1).

C 1s

Fig. S2c shows the C 1s region of the AP-XPS spectra. The dominant peak is due to hydrocarbon contaminants. Other small peaks at higher positions are due to oxygenated hydrocarbons, either as contaminants or as reaction products. Positions of all the peaks depend on the experimental conditions in a similar way to the ceria related peaks in the Ce 4d and O 1s regions. The intensity of the oxygenated hydrocarbon peaks is highest when both CO_2 and H_2 are present, which could indeed be due to the production of various CO_2 hydrogenation reaction products.

Section S3 - Details of GIXS analysis and fits along q_r=0

Fig. S3 shows an example of a line profile along $\mathbf{q_r}$=0 with explanation.

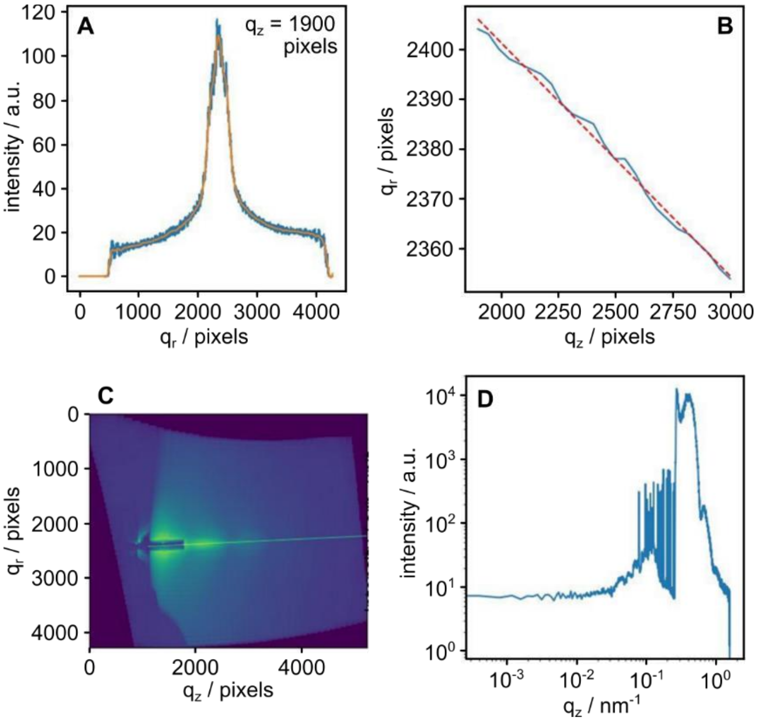

Fig. S3. Line profile analysis along $\mathbf{q_z}$ at $\mathbf{q_r}$ = 0. To account for the sample tilt, a vertical line profile was taken along a slanted line. The center of the sample at each point in the scattering map was determined using a Python code, identifying the maximum intensity within 50-pixel-wide profiles from 1900 to 2900 pixels, where the beamstop is absent. **(a)** An example of a maximum intensity identification at a $\mathbf{q_z}$ of 1900 pixels. Due to the absence of a structure factor, the strongest signal at any given $\mathbf{q_z}$ is always perpendicular to the origin. **(b)** The maxima positions along the horizontal plane were fitted with a simple linear fit. The resulting line was extrapolated from the $\mathbf{q_z}$ at origin to $\mathbf{q_{max}}$. Because of the overlap of ceria cylinders, as shown in the AFM image, no discernible in-plane form factor is observable. **(c** and **d)** Example of a raw GIXS image and the vertical line profile.

Due to the absence of an in-plane form factor, the vertical line profile was fitted using a cylinder with a radius five times greater than its height. Surface roughness was modeled using a surface fractal approximation, where the total fractal enclosing circle had a diameter of 15 nm and consisted of spherical units ranging from 0.5 to 2 nm in diameter. The fractal form factor was multiplied by the cylinder form factor. To ensure consistency, the scattering multiplier was kept constant across samples within the same measurement series, while height, polydispersity, scattering length density (SLD), fractal dimensionality, unit cell diameter, and background were varied. Due to uncertainties in the total reflected light intensity and substrate reflectivity, the obtained SLD is unitless and only comparable within the same series.

All the raw data (after converting pixels to $\mathbf{q}$) and the fits are presented in Figs. S4-5.

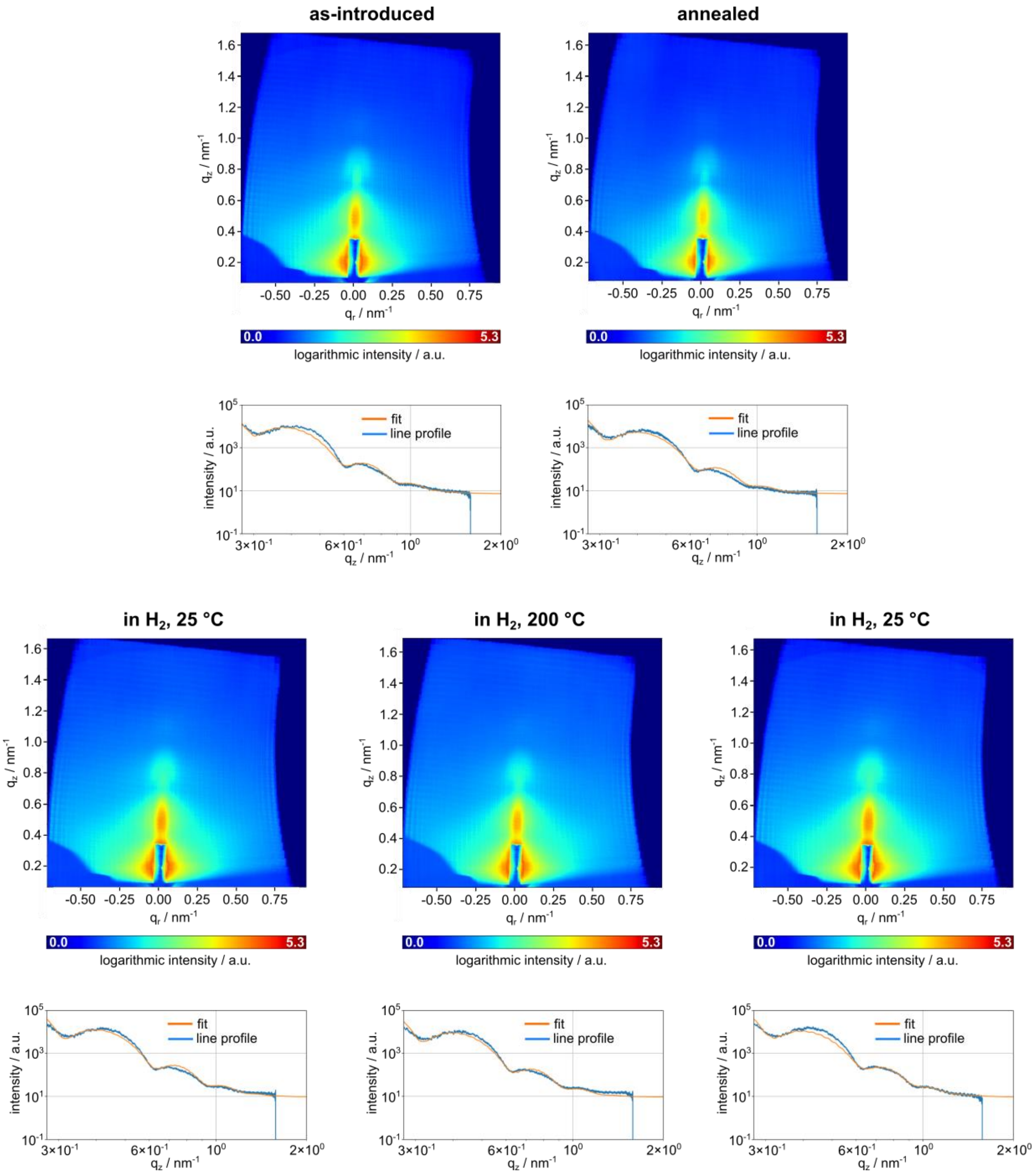

Fig. S4. Scattering images and fits of the line profiles for experimental set 1.

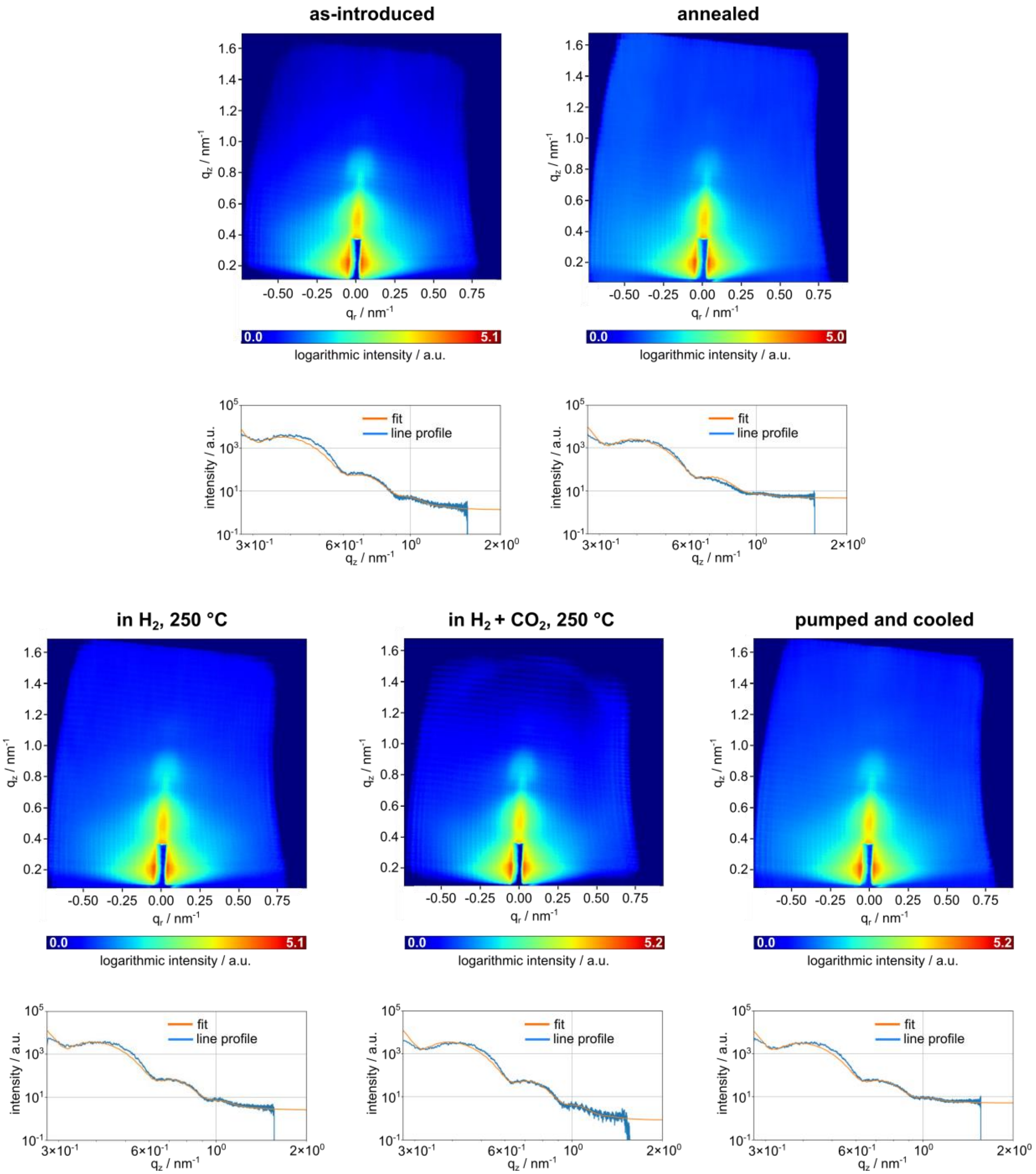

Fig. S5. Scattering images and fits of the line profiles for experimental set 2.

Section S4 - X-ray reflection critical angle analysis

The details of the X-ray reflection critical analysis are presented in Fig. S6. We used the following formula to extract the critical angle:

$$2\theta = arcsin(q_z/(4 * \pi/\lambda)) * 360°/\pi$$

As an example, θ and q_z in a table for the first experimental set:

	SiO_2	CeO_x	SiO_2	CeO_x
as prepared	0.134	0.191	1.51524	2.15985
annealed	0.15	0.207	1.69618	2.3408
H_2 25 °C	0.147	0.205	1.66225	2.31818
H_2 200 °C	0.15	0.212	1.69618	2.39735
H_2 afterwards	0.137	0.212	1.54917	2.39735

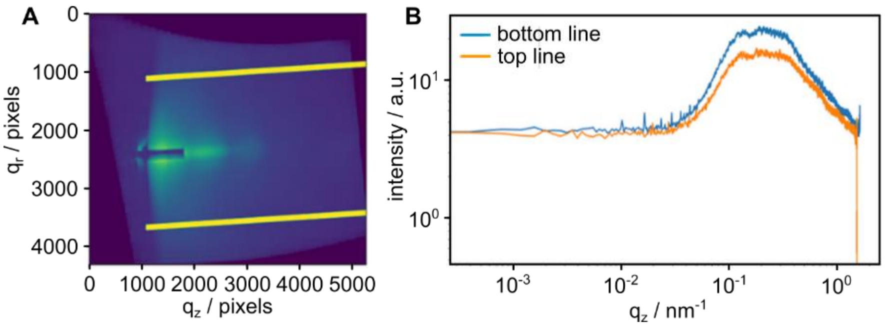

Fig. S6. **(a** and **b)** Line profiles for determining the critical angle of X-ray reflection. Reflectivity was measured at high $\mathbf{q_r}$ values to minimize the influence of the form factor on the critical intensities near the Yoneda line. The vertical lines were tilted according to the estimated sample tilt obtained from the method described above (Fig. S3). To account for sample inhomogeneities, we averaged the top and bottom lines during evaluation (Figs. S7-8). The line width was set to 50 pixels due to the low signal strength at high $\mathbf{q_r}$.

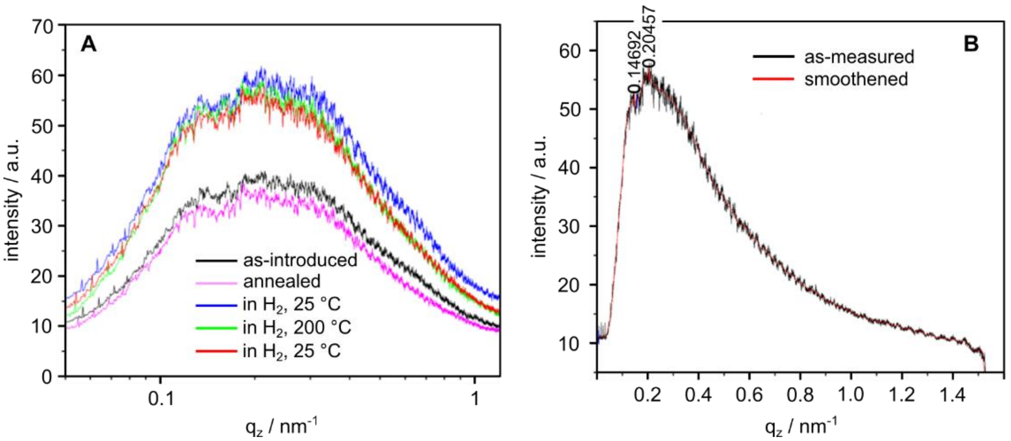

Fig. S7. X-ray reflection line profiles – first set. **(a)** Line profiles at various conditions. **(b)** After smoothing, one of the line profiles. The maxima are indicated on the plot, which are converted to the critical angle.

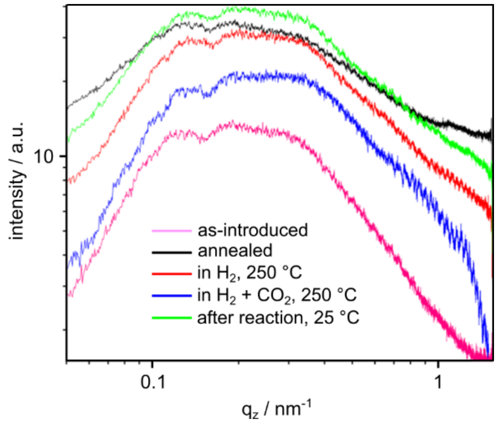

Fig. S8. X-ray reflection line profiles – second set. Line profiles at various conditions. A third maximum, which is present in some of the line profiles, is not due to the critical angle but the form factor.

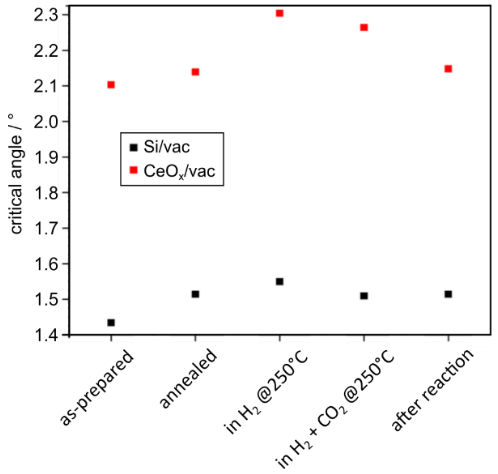

Fig. S9. Critical angles of X-ray reflection of the second set of experiments. In the main text, they are represented as difference between each other to account for contamination.

Section S5 - Explanation of scattering models and used formulas

To fit the vertical ($\mathbf{q_r} = 0$) line profiles, a model was used that combines oriented cylinders at an incidence angle of 88.5° with a fractal function, a background term, and SLD. For simplicity, a constant sample volume was assumed, as the variations in volume fraction between profiles are either negligible or directly related to material density changes due to oxidation and hydrogen incorporation. Due to the complex sample geometry, uncertainties in beam intensity and azimuthal alignment, the exact oxidation state of the cylinder core, and residuals from the patterning process (such as incomplete liftoff, cylinder merging, and carbon contamination), the evaluated densities, roughness, and polydispersity are unitless. Therefore, these parameters should be interpreted only in the context of understanding the sample's evolution under different conditions.

Cylinder form factor:

$$F(q,\alpha) = 2(\Delta\rho)V \frac{sin(\,(1/2)\,qL\,cos\,\alpha\,)}{(1/2)\,qL\,cos\,\alpha} \frac{J_1(qR\,sin\,\alpha)}{qR\,sin\,\alpha}$$

where J1 is the first-order Bessel function, L represents the cylinder length, $\Delta\rho$ is SLD of cerium oxide as a function the used photon energy (1000 eV), V is the volume of the cylinder, and R denotes the radius. Here, α is the angle between the cylinder axis and the scattering vector **q.**

We used the BornAgain software to perform a sanity check of our assumptions (S9). We show these sanity checks as a series of figures (Figs. S10-S17) and discuss them below.

When compared to the cylinder form factor with a well-defined diameter, the measured out-of-plane profiles do not exhibit sharp minima. Additionally, the in-plane scattering of the sample does not feature any minima. This can only be explained by the undefined and random spatial shape of the cylinders. The randomness arises from the template used to create a random cylinder positioning, leading to cylinder overlapping and merging. In addition, irregularities introduced during the liftoff process enhance this effect further. These irregularities are highlighted in the AFM measurements. Since the in-plane size of the cylinders is fully random and continuous, its contribution to the out-of-plane scattering does not produce distinct minima (Fig. S10). Instead, it influences the slope of the 2D form factor. This behavior can be approximated by highly polydisperse cylinders with a mean diameter exceeding the upper resolution limit. To incorporate this in the analytical solution, the radius R in the formula is defined as L×5, with the standard deviation of R, σ_R, set to σ_L×5. However, this assumption, along with the reflectivity from the substrate, results in a steeper slope than what is observed in the experimental data (Fig. S11). Another factor must therefore be considered. Introducing a core within the cylinder with a higher or lower density allows for variation in the slope (Fig. S12). However, the experimentally observed slope cannot be fully explained by any reasonable density contrast between the core and shell. Changes in substrate reflectivity and cylinder coverage can also affect the form factor slope (Fig. S13-S14), but these factors vary only minimally between samples and can be considered constant. The best-fitting simulation, both in terms of slope and overall appearance, consists of a random assembly of core–shell cylinders with a denser core, a highly polydisperse cylinder radius, and a low polydisperse height. These are decorated with randomly oriented smaller spherical nanoparticles, with a shell-like density and an approximate size of 2.5 nm (Fig. S15). This nanoparticle decoration mimics the grain structure of the CeO_x cylinders observed in the AFM image and can be interpreted as surface roughness. Due to the extensive parameter space associated with the sample geometry, fitting the scattering curves for all conditions using simulations is computationally impractical. Instead, a fractal function was used to align the slope of the analytical solution for out-of-plane scattering with the experimental data:

$$S(q) = 1 + \frac{D_f \Gamma(D_f - 1)}{[1 + \frac{1}{(q\xi)^2}]^{(D_f-1)/2}} \frac{sin\,[(D_f - 1)tan^{-1}(q\xi)]}{(qR_0)^{D_f}}$$

where:

D_f is the fractal dimension,

$\Gamma(D_f - 1)$ is the Gamma function evaluated at $D_f - 1$

q is the scattering vector,

ξ is the correlation length,

R_0 is a characteristic length scale of unit cell.

This function incorporates all the previously mentioned influences on the slope as a dummy function. The best fit for ξ was found at 15 nm, suggesting that the top 15 nm of the cylinders may be highly porous, while the fixed bottom remains well-defined. This value was kept constant, while D_f and R_0 were varied to match the changing slope under different conditions. Given the negligible influence of sample height and diameter on the slope (Figs. S10, S16), as well as the constant incident angle, coverage, and substrate density, the variation in these two parameters reflects a relative change in the surface roughness of the CeO_x cylinders. This roughness may include features such as pockets, holes, and channels. Since optical density of ceria cylinders only affects the overall scattering intensity (Fig. S17) without altering the slope, its relative change during treatment is directly reflected in the obtained SLD. It is worth noting that the simulation suggests a core–shell structure, which was omitted from the analytical solution for simplicity. The relative density changes derived from scattering are primarily influenced by the core density, whereas the oxidation state measured via the XPS mainly originates from the shell.

Finally, the background contribution accounts for Compton scattering from introduced gases, carbon contamination, and background radiation from the heater under different conditions. The surface-fractal term is an effective descriptor of the $\mathbf{q_z}$ decay and thus subsumes contributions from unresolved morphology and residual geometry/background differences. Consequently, the fitted fractal dimensionality is robust for relative comparisons within a contiguous measurement series (and not cross-set).

BornAgain simulation vertical line profiles at the origin were used to rationalize the underlying assumptions. All BornAgain simulations assume perfectly geometric cylinders on a single-density substrate with a 1000 eV beam energy. The detector width spans from −9° to 9° horizontally and from 0° to 18° vertically, using a spherical detector. No beam distribution is considered with the incident angle fixed at 1.6°. The cylinders are randomly distributed, and the densities in these rationalization simulations are not calibrated to material references. The simulations serve only to illustrate trends in parameter variations and their effects. We only present here one of them for comparison purposes (Fig. S15).

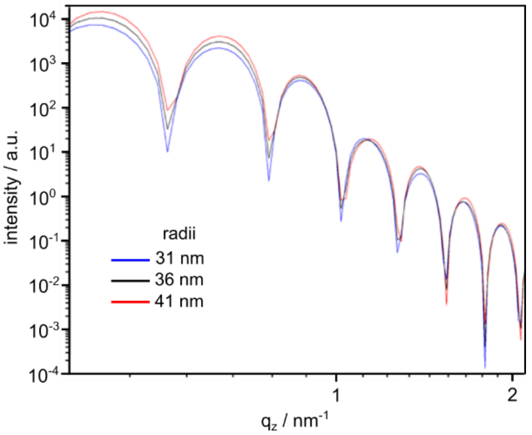

Fig. S10. Scattering analysis plot #1. Variation of the cylinder radius with a standard deviation of 20 nm, shows that cylinder overlapping and merging cannot result in changes in minima position in our conditions. The height is fixed to 24.5 nm and the incident angle is fixed to 1.6°.

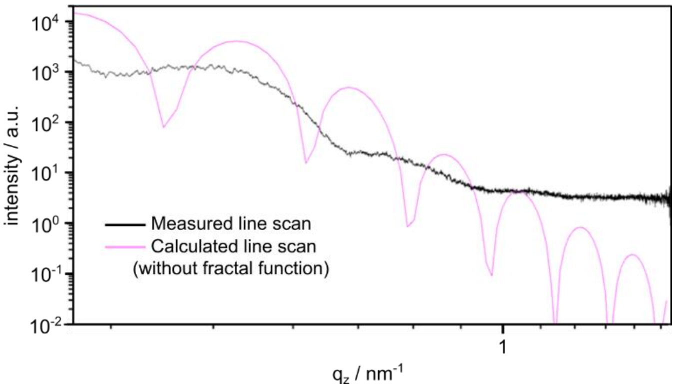

Fig. S11. Scattering analysis plot #2. Comparison of a calculated line scan from the form factor generated by 24.5 nm high cylinders with a mean diameter of 74 nm and a standard deviation of 25 nm (without a fractal function) and the measured out-of-plane scattering curve.

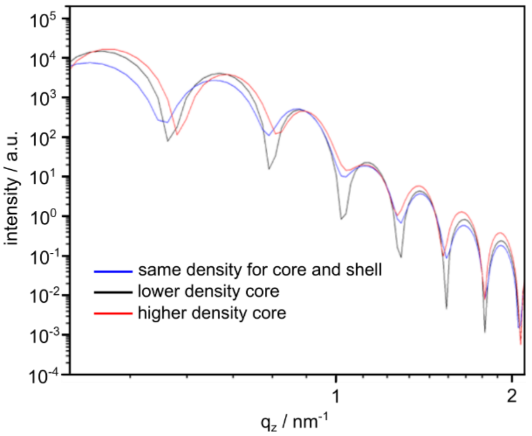

Fig. S12. Scattering analysis plot #3. Comparison of full cylinders and core-shell cylinders, where "low density core" means the core has 0.667 times the density of the shell, and "high density core" means the core has 1.5 times the density of the shell. The height is fixed to 22 nm and the diameter is fixed to 34 nm.

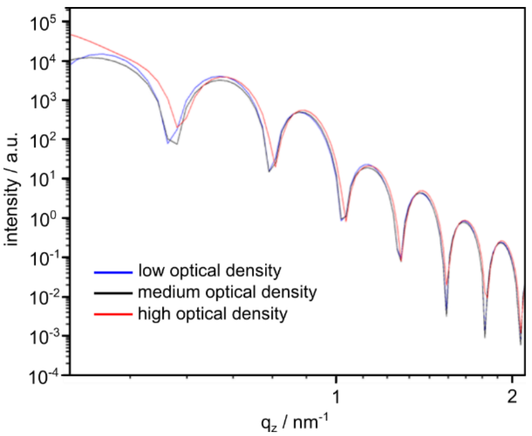

Fig. S13. Scattering analysis plot #4. Comparison of the scattering slope at 1.6° incident angle for a substrate with optical densities of 1/100, 1/10, and 1/3.

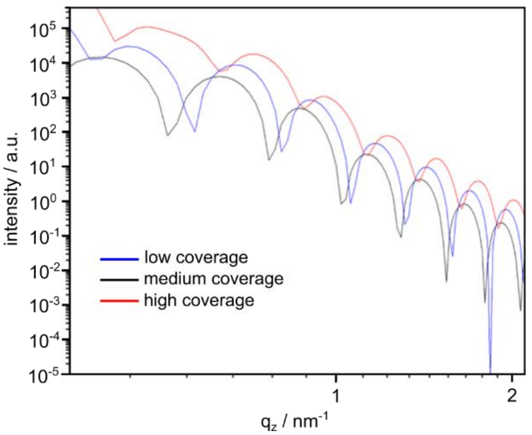

Fig. S14. Scattering analysis plot #5. Comparison of the different coverage of cylinders. Mean diameter is fixed to 36 nm with a standard deviation of 20 nm, height fixed at 24.5 nm. Coverage is set as 10^{-5} nm^{-2}, 2×10^{-5} nm^{-2} and 5×10^{-5} nm^{-2}. Even with significant differences in coverage, there is no major difference between the slopes.

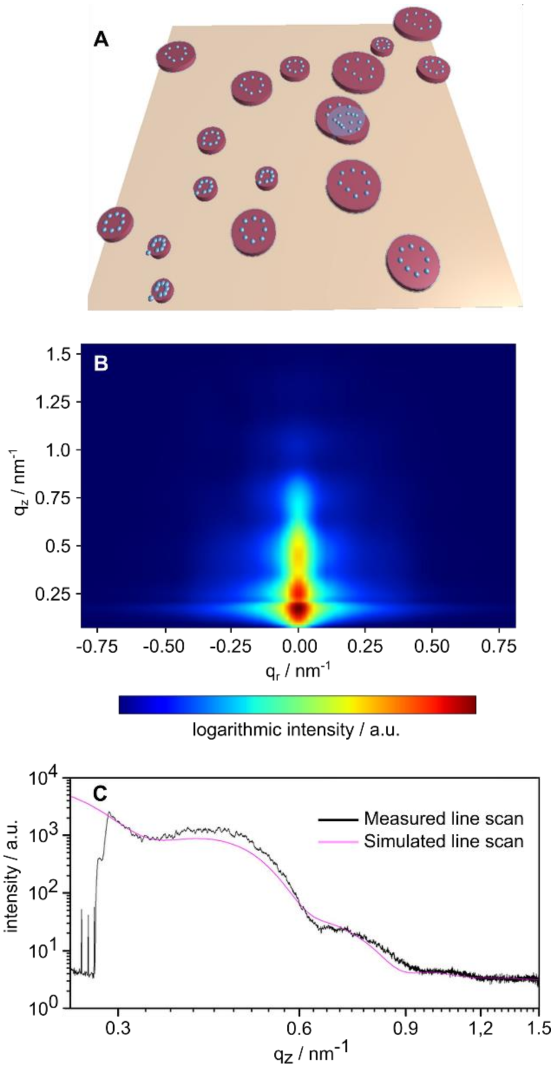

Fig. S15. Example of a simulated image and analysis. A simulated scattering pattern is shown in **(b)**, obtained using a simulated sample geometry in **(a)**. **(c)** Comparison of line profiles between the as-prepared sample and the simulation.

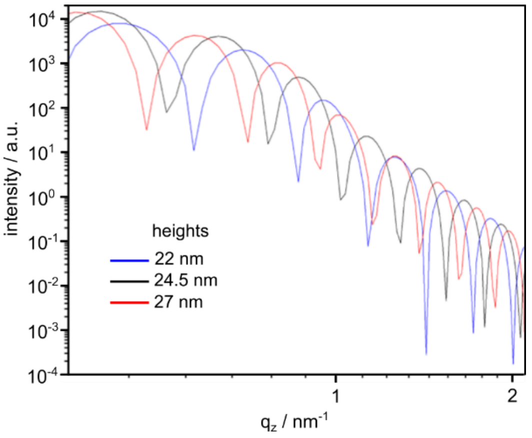

Fig. S16. Scattering analysis plot #6. Variation of disk height in nm. Mean diameter is fixed to 36 nm with a standard deviation of 20 nm, and the incident angle fixed to 1.6°.

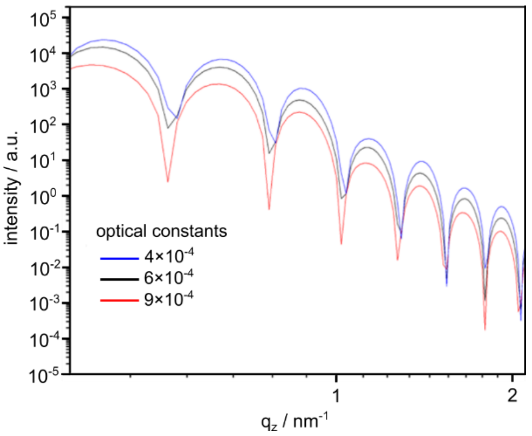

Fig. S17. Scattering analysis plot #7. Result of changing the optical constants of the simulated cylinders while keeping all other parameters constant.

Section S6 - Further analysis of the trends

Fig. S18 shows other results of the fits of XPS and GIXS data. The top row shows the percentage of the Ce^{4+} related peaks (two red peaks in Figs. S2a and S2d) in the Ce 4d region, excluding the silica related peaks. The trend observed in Fig. S18-i is very similar to that of Fig. 2-i. This shows that although the linear combination approach is a cruder approach, it does not fail to catch the general trends observed in the chemical state of the surface. The fitting approach is more precise, but it cannot be used to estimate the ratio of the Ce^{4+} cations, because of the yellow features that have a contribution from Ce^{4+} and Ce^{3+} cations.

Fig. S18-ii shows the FWHM of the oxide peak in the O 1s region. For the H_2-only experiment, after annealing in high vacuum, the oxidation state of ceria is roughly half 4+ and half 3+. This is why the FWHM is highest as two convoluted peaks with slightly different binding energies are produced. In H_2 at 25 °C, FWHM is lowered as Ce^{4+} is favored overed Ce^{3+}. 200 °C in H_2 is a unique case, because some Ce^{4+} species are converted to Ce^{3+} via CeO_2H_y formation which should generate a different ceria-related peak in the O 1s region. Therefore, the FWHM is large again. As we mentioned in the main text, in the literature, subsurface/bulk CeO_2H_y formation was observed above 327 °C. Difference in the current study should be due to the defective nature of the polycrystalline thin film allowing hydrogen diffusion. Due to a similar reason, hydride formation is also more pronounced in our studies. In the second set of experiments, the initial FWHM of the oxide peak is similar to the first set, and annealing in H_2 increases it, which should be due to oxyhydroxide formation. Once CO_2 is added, some of the oxyhydroxides are converted to 4+ species, hence lower FWHM.

The SLD trends presented in Fig. S18-iii, show some resemblance to the critical angle trends. As we mentioned, critical angle is a better descriptor of the electron density because SLD can be affected by the contaminants. Critical angle of X-ray reflection is also affected by the contaminants, but under the assumption that contaminants affect the reflection of silica and ceria similarly, relative critical angle should be a reliable descriptor of electron density changes in ceria.

Finally, as mentioned in the main text, the changes in the height (bottom row of Fig. S18) do not show any significance in different conditions.

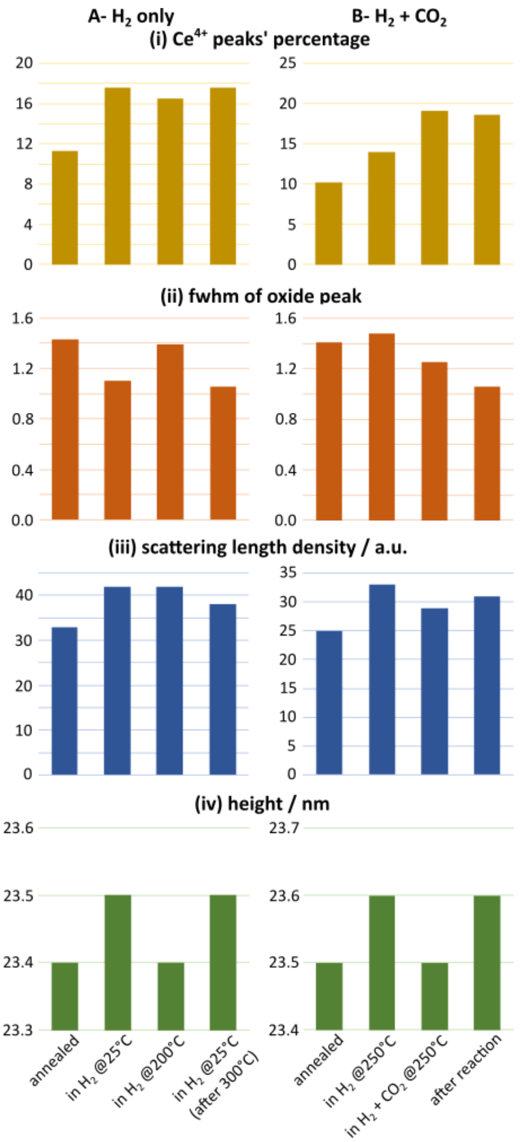

Fig. S18. Other trends obtained from the XPS and GIXS data. The top two rows are from the analysis of the XPS data. Third row, SLD of ceria cylinders, obtained from the $\mathbf{q}_r$=0 line profile. The last row, height of ceria cylinders, obtained from the fit to the central region of the $\mathbf{q}_r$=0 line profile.

Section S7 - Effect of initial annealing

The effect of initial annealing is not discussed in the main text. Its primary role is to reduce the sample surface, as detailed in our previous study (S1). However, scattering analysis reveals two additional major effects (Fig. S19): a ~1 nm decrease in height and a significant increase in polydispersity. We attribute the height reduction to the initial low packing density of the ceria film (S10). Annealing increases the packing density, making the structure more compact, which leads to both a decrease in height and reduced roughness (1st and 2nd plots in Fig. S19). A similar height reduction is observed in the second set of experiments, suggesting that hydrogen incorporation and its subsequent removal in air create a porous structure that is partially restored upon annealing at 450 °C. The increase in height polydispersity upon annealing (3rd plot in Fig. S19) is also noteworthy and likely linked to the enhanced compactness of the nanostructure. One possible explanation is that only a subset of the cylinders undergoes shrinkage during annealing, leading to greater height variation. We should also mention that annealing decreases contaminant coverage (from storage in air), which could also affect both the height and its polydispersity.

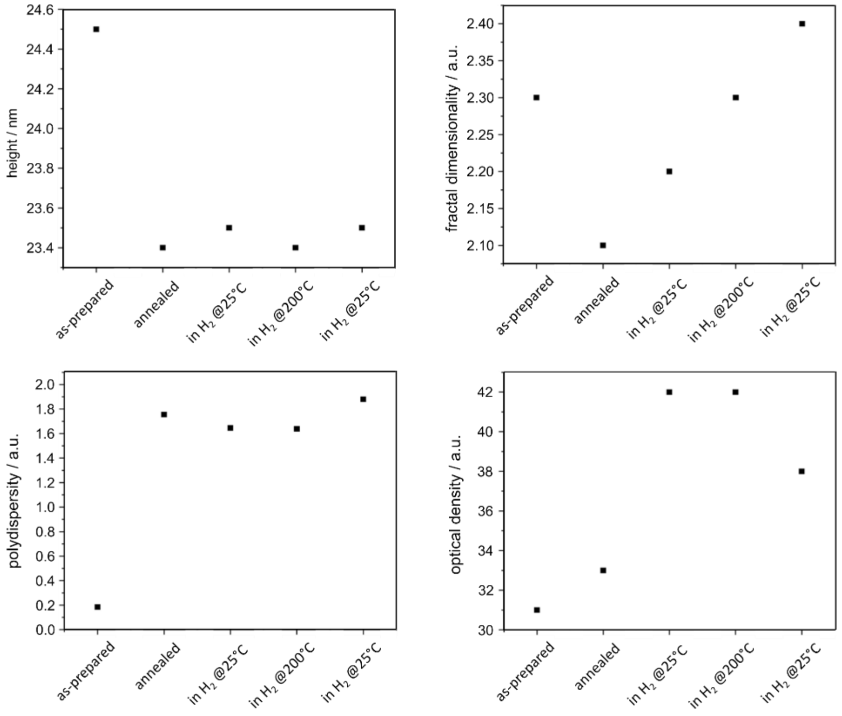

Fig. S19. Trends of the scattering analysis of the first set of experiments including the as-prepared data point.

References for the supplementary information

(S1) A. Ben Yaacov et al., Oxidation and reduction of polycrystalline cerium oxide thin films in hydrogen. J. Phys. Chem. Lett. 14, 7354–7360 (2023).

(S2) M. Baron, O. Bondarchuk, D. Stacchiola, S. Shaikhutdinov, H. J. Freund, Interaction of gold with cerium oxide supports: CeO_2(111) thin films vs CeO_x nanoparticles. J. Phys. Chem. C 113, 6042–6049 (2009).

(S3) K. I. Maslakov et al., XPS study of ion irradiated and unirradiated CeO_2 Bulk and thin film samples. Appl. Surf. Sci. 448, 154–162 (2018).

(S4) D. R. Mullins, S. H. Overbury, D. R. Huntley, electron spectroscopy of single crystal and polycrystalline cerium oxide surfaces. Surf. Sci. 409, 307–319 (1998).

(S5) V. Matolín, et al., Water interaction with CeO_2(111)/Cu(111) model catalyst surface. Catalysis Today 181, 124–132 (2012).

(S6) Z. R. Li et al., Oxidation of reduced ceria by incorporation of hydrogen. Angew. Chem., Int. Ed. 2019, 58, 14686–14693.

(S7) Z. R. Li et al., Interaction of hydrogen with ceria: Hydroxylation, reduction, and hydride formation on the surface and in the bulk. Chem. Eur. J. 27, 5268–5276 (2021).

(S8) Y. Lykhach et al., Water chemistry on model ceria and Pt/ceria catalysts. J. Phys. Chem. C 116, 12103−12113 (2012).

(S9) G. Pospelov et al., BornAgain: software for simulating and fitting grazing-incidence small-angle scattering. J. Appl. Cryst. 53, 262–276 (2020).

(S10) G. Atanassov, R. Thielsch, D. Popov, Optical properties of YiO_2, Y_2O_3, and CeO_2 thin films deposited by electron beam evaporation. Thin Solid Films 223, 288–292 (1993).
